# Improved Graph Embedding for Robust Recognition with outliers

**DOI:** 10.1038/s41598-018-22207-x

**Published:** 2018-03-09

**Authors:** Peiyang Li, Weiwei Zhou, Xiaoye Huang, Xuyang Zhu, Huan Liu, Teng Ma, Daqing Guo, Dezhong Yao, Peng Xu

**Affiliations:** 1 The Clinical Hospital of Chengdu Brain Science Institute, MOE Key Lab for Neuroinformation, University of Electronic Science and Technology of China, Chengdu, China; 2 School of life Science and technology, center for information in medicine, University of Electronic Science and Technology of China, Chengdu, China

## Abstract

Artifacts in biomedical signal recordings, such as gene expression, sonar image and electroencephalogram, have a great influence on the related research because the artifacts with large value usually break the neighbor structure in the datasets. However, the conventional graph embedding (GE) used for dimension reduction such as linear discriminant analysis, principle component analysis and locality preserving projections is essentially defined in the L2 norm space and is prone to the presence of artifacts, resulting in biased sub-structural features. In this work, we defined graph embedding in the L1 norm space and used the maximization strategy to solve this model with the aim of restricting the influence of outliers on the dimension reduction of signals. The quantitative evaluation with different outlier conditions demonstrates that an L1 norm-based GE structure can estimate hyperplanes, which are more stable than those of conventional GE-based methods. The applications to a variety of datasets also show that the proposed L1 GE is more robust to outlier influence with higher classification accuracy estimated. The proposed L1 GE may be helpful for capturing reliable mapping information from the datasets that have been contaminated with outliers.

## Introduction

The technological advances in data acquisition and storage result in a large number of high-dimensional and ultra-high-dimensional datasets in various biomedical applications^1^, such as electroencephalogram (EEG), gene expression, and sonar imaging. These datasets might be redundant and result in the curse of dimensionality or excessive computational consumption when they are utilized for model construction and pattern recognition, although they are certainly desirable for modeling biological processes^2^. This issue promotes the utilization of dimension reduction technologies such as principle component analysis (PCA), linear discriminant analysis (LDA), and locality preserving projections (LPP). In essence, these linear or nonlinear projection strategies can be unified in the structure of graph embedding (GE), which constructs a graph space based on the affinity information between each pair of samples and represents each vertex of the graph with a low-dimensional vector that preserves similarities between the vertex pairs^3^. Since the establishment of this framework, there have been varieties of successful applications in biomedical signal analysis^4–6^.

However, in data analysis, such as biomedical research, a great challenge exists, i.e., the inevitable artifacts in the signal recordings^7,8^, which are one of the major factors accounting for reduced signal quality. Artifacts can be caused by various factors such as the clinical image artifacts from illumination variations and dust particles, measurement errors in biochemistry^8^, independent scatters caused by biological tissues that are smaller than the acoustical wavelength^9^ and artifacts in EEG recordings due to blinks and eye movement, a large number of spontaneous brain activities, or spikes. Artifacts are usually characterized by several orders of magnitude larger than the signal of interest, which cannot be described by the standard Gaussian distribution. Thus, when using the conventional L2 norm-based approaches like maximum-likelihood (ML) and least squares to estimate the noise variance or to extract features, the results are usually inevitably biased by the components that express the outliers^9–11^. To resist the noise influence, Cai *et al*.^12^ redefined kernel discriminant analysis with spectral graph analysis (SRKDA) and estimated the projections in a more efficient and robust way. Though a series of improvements^13–15^ have been proposed to widen the applications of GE extensions in recent years, few of them have focused on artifact restriction.

The easiest way to depress the influence of artifacts for further analysis is to reject the contaminated samples. However, this is not favorable because it may lead to the loss of other useful information. A more favorable way is to detect the artifacts or outliers and then use approaches like regression, the blind source separation (BSS) method for artifact removal. Although some methods have been proposed to find outliers, it is difficult to determine the extent to which the peculiarities would be outliers^8^. Even if the artifacts or the outliers are located, the process of eliminating the artifact component by using BSS methods such as independent component analysis (ICA) is not trivial and may result in the loss or distortion of useful information in the extracted features^16^. To avoid the loss of data while restricting the outlier influence for feature extraction, one of the most efficient ways is to extend the conventional L2 norm-based models to the Lp (p ≤ 1) norm space. For example, Kwak *et al*. translated the L2 norm structure of conventional PCA with the L1 norm to restrict the outlier influence for dimensional reduction^17^. Wang *et al*. proposed replacing the covariance matrix with the L1 norm structure in the Rayleigh quotient expression to improve the robustness of common spatial patterns (CSP) toward outliers for a motor imagery-based brain computer interface (BCI)^11^. In our previous conference work^18^, Zhou developed the spectral regression (SR) in the L1 norm space to obtain robust parameters under outlier conditions while neglecting the possible outlier effects on the estimation of response vectors in graph embedding. Similar to Zhou’s work, Nie *et al*. measured the distance between any pair of projected vertices in the L1 norm space^19^. To facilitate the solution, they still restrained the denominator of the Laplacian embedding in the L2 norm space. Accordingly, this may also inevitably be influenced by outliers. In this work, we propose a novel graph-embedding framework based on the L1 norm maximization strategy to restrict the outlier influence in dimensionality reduction, which is usually encountered in biological signals.

## Results

In recent years, graph embedding and its extensions have gained more and more attention and contributed to a series of relative reports. Among these, discriminant analysis-based extensions, such as RLDA, HLDA and NDA, and spectral regression-based extensions, such as SRDA, SRKDA and local preserving projection and its extensions, are commonly mentioned and have been successfully applied to both engineering technology and neural science research^12,20–25^. These methods have their own superiorities when dealing with different data problems such as noise and heteroscedasticity in dimension reduction^3,26^. In addition to these GE extensions, other L1 norm-based linear classifiers such as Lasso SRDA, L1 SR and L1 LDA have also been proposed to resist outlier influence in recent years. Thus, in this section, we will investigate the performance difference between our proposed L1 GE algorithm and these popular classifiers for classification tasks. Classification accuracy is adopted as the performance index for evaluation. All of our experiments were performed on a Core i3 3.30-GHz Windows 7 machine with 8 GB of memory.

We mainly investigate the performance difference between L1 GE and the 10 other popular classifiers (7 GE extensions and 3 L1 norm-based linear classifiers), i.e., LDA^27^, Regularized LDA (RLDA)^28^, Heteroscedastic LDA (HLDA)^29^, Non-parameter LDA (NDA)^30^, Spectral regression discriminant analysis (SRDA)^31^, LPP^32^, SRKDA^12^, Lasso SRDA^31^, L1 LDA^33^ and L1 SR^18^, for dimension reduction and feature extraction. In both simulation and real datasets experiments, we simply set the regularization parameter for RLDA and SRDAs (i.e., SRDA, SRKDA and Lasso SRDA) as 1.0 and 0.7 according to^31^. As the types of kernels were not what we considered in this work, we just used the Gaussian kernel for SRKDA as reported in^12^. In this work, we tuned the kernel width parameter σ in SVM to achieve the best testing performance for SVM. Then, the same kernel width parameter σ was used for SRKDA. The weights estimation for LPP is straightforward according to^32^. The 3 neighbors in NDA were used as reported in a previous study^30^.

### Simulation studies

In this section, two classes from a 2-D Gaussian distribution with different means are adopted^34^. Class 1 is from a Gaussian distribution with mean (3.00, 3.00) and variance (0.5, 0.5), and class 2 is from a Gaussian distribution with mean (1.85, 1.85) and variance (0.5, 0.5). Theoretically, the optimal hyperplane (i.e., decision boundary) for differentiating the two classes should be along 135 degrees. If the outliers are introduced, the corresponding classification hyperplane may be biased. During simulation, the training set consisted of 220 samples, with each class being 110 samples, and the testing set contained the same label distribution as the training set, i.e., 110 samples for each class in the testing set.

#### Effect of outlier occurrence rate

**Figure 1:**
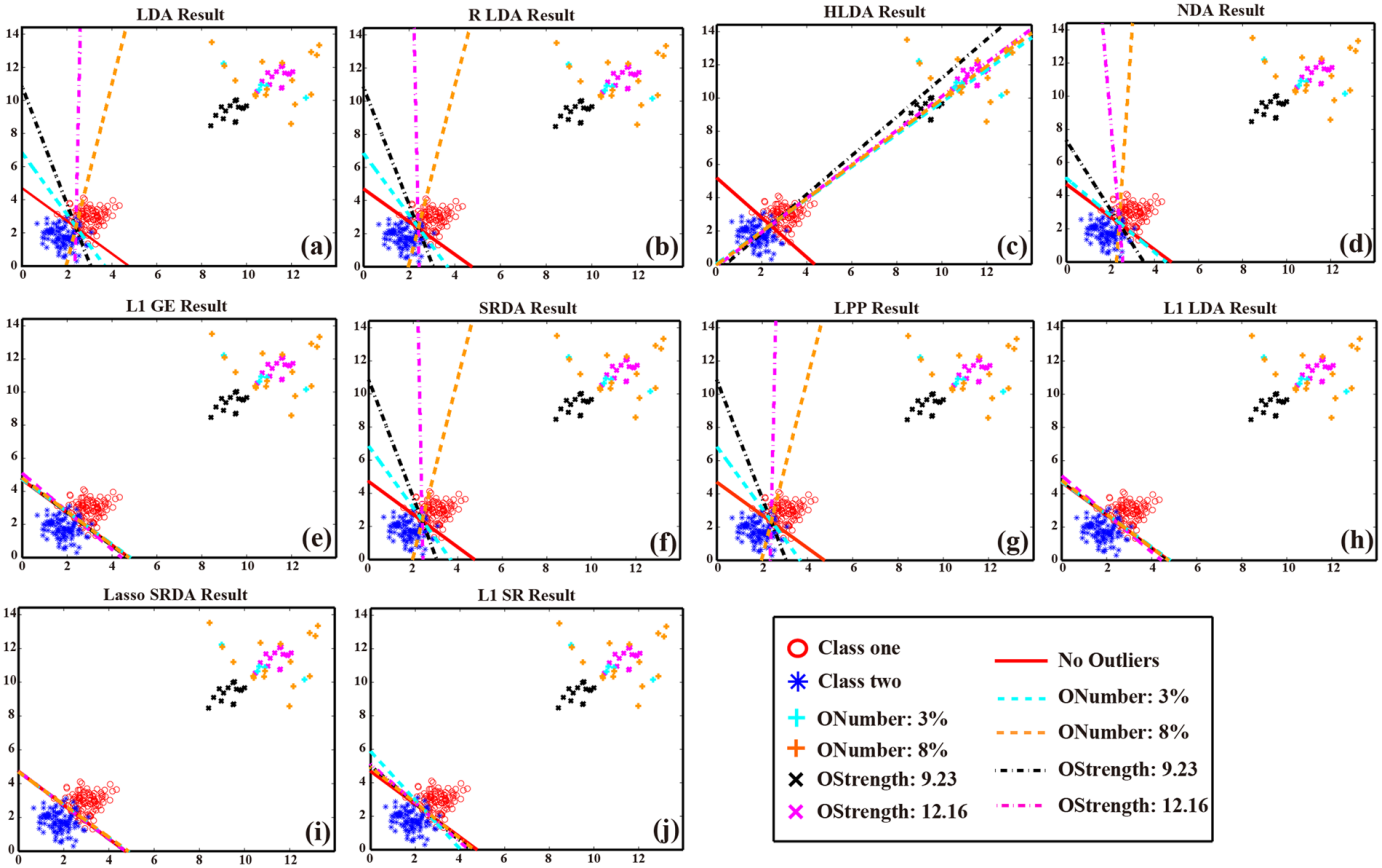
The effect of outliers on decision boundaries. (**a**) LDA. (**b**) RLDA. (**c**) HLDA. (**d**) NDA. (**e**) L1 GE. (**f**) SRDA. (**g**) LPP. (**h**) L1 LDA. (**i**) Lasso SRDA. (**j**) L1 SR.

This simulation generates datasets contaminated by different numbers of outliers. The outlier is generated from the Gaussian distribution with mean (13.65, 13.65) and variance (0.5, 0.5). The number of outliers is set as 0%, 3% and 8% of the sample number. In each outlier condition, the outliers are evenly assigned to the two classes and the procedure is repeated 100 times. The mean accuracies are reported in Table 1 on the left side. The corresponding mean effect of the outlier number on the hyperplane is visually given in Fig. 1 for all the linear classifiers. As SRKDA constructs the hyperplane in the kernel space that is essentially different from the original data space, the corresponding hyperplane of SRKDA is not presented in Fig. 1 under different outlier occurrence rates.

**Table 1:** Classification accuracy and the projection direction in different outlier conditions.

Methods	Occurrence rate (%)			Occurrence Strength		
	0%	3%	8%	0.00	${\bf{6.00}}{\boldsymbol{\times }}\sqrt{{\bf{2}}}$	${\bf{8.60}}{\boldsymbol{\times }}\sqrt{{\bf{2}}}$
LDA (Angle)	**0**.**96 ± 0**.**01 (135**.**10 ± 4**.**38)**	0.91 ± 0.03 (128.85 ± 32.27)	0.73 ± 0.06 (79.89 ± 51.75)	**0**.**96 ± 0**.**01 (135**.**10 ± 4**.**38)**	0.88 ± 0.04 (113.72 ± 50.08)	0.78 ± 0.04 (90.36 ± 57.35)
(Time/s)	0.62	0.64	0.61	0.62	0.61	0.63
R LDA (Angle)	**0**.**96 ± 0**.**01 (135**.**09 ± 4**.**31)**	0.91 ± 0.03 (128.93 ± 32.10)	0.73 ± 0.06 (79.90 ± 51.79)	**0**.**96 ± 0**.**01 (135**.**09 ± 4**.**31)**	0.88 ± 0.03 (113.25 ± 38.82)	0.78 ± 0.05 (92.08 ± 53.09)
(Time/s)	0.64	0.65	0.64	0.64	0.64	0.64
H LDA (Angle)	**0**.**96 ± 0**.**02 (134**.**43 ± 16**.**47)**	0.53 ± 0.05 (45.02 ± 1.54)	0.51 ± 0.04 (44.63 ± 1.07)	**0**.**96 ± 0**.**02 (134**.**43 ± 16**.**47)**	0.62 ± 0.14 (52.53 ± 30.37)	0.53 ± 0.08 (47.19 ± 16.78)
(Time/s)	2.80	2.83	2.82	2.80	2.83	2.84
NDA (Angle)	**0**.**96 ± 0**.**01 (135**.**15 ± 5**.**65)**	0.94 ± 0.04 (133.15 ± 28.20)	0.74 ± 0.05 (85.12 ± 53.66)	**0**.**96 ± 0**.**01 (135**.**15 ± 5**.**65)**	0.90 ± 0.04 (125.07 ± 52.00)	0.79 ± 0.05 (95.47 ± 52.32)
(Time/s)	4.56	4.58	4.56	4.56	4.56	4.58
SRDA (Angle)	**0**.**96 ± 0**.**01 (135**.**09 ± 4**.**33)**	0.91 ± 0.06 (128.23 ± 51.50)	0.73 ± 0.06 (79.41 ± 54.41)	**0**.**96 ± 0**.**01 (135**.**09 ± 4**.**33)**	0.88 ± 0.03 (113.27 ± 38.85)	0.78 ± 0.05 (92.87 ± 53.10)
(Time/s)	0.82	0.83	0.83	0.82	0.82	0.82
LPP (Angle)	**0**.**96 ± 0**.**01 (135**.**10 ± 4**.**38)**	0.91 ± 0.08 (128.33 ± 51.75)	0.73 ± 0.03 (75.74 ± 53.71)	**0**.**96 ± 0**.**01 (135**.**10 ± 4**.**38)**	0.88 ± 0.03 (114.70 ± 42.09)	0.78 ± 0.05 (90.16 ± 53.11)
(Time/s)	**1**.**77**	1.75	1.70	**1**.**77**	1.72	1.74
SRKDA	0.74 ± 0.20	0.73 ± 0.18	0.74 ± 0.19	0.74 ± 0.20	0.74 ± 0.19	0.74 ± 0.19
(Angle)	—	—	—	—	—	—
(Time/s)	0.97	1.00	0.95	0.82	0.96	0.94
L1 LDA (Angle)	**0**.**96 ± 0**.**01 (134**.**70 ± 5**.**11)**	**0**.**95 ± 0**.**02 (134**.**03 ± 12**.**72)**	**0**.**79 ± 0**.**03 (134**.**74 ± 13**.**65)**	**0**.**96 ± 0**.**01 (134**.**70 ± 5**.**11)**	**0**.**92 ± 0**.**02 (135**.**07 ± 9**.**04)**	**0**.**81 ± 0**.**03 (136**.**45 ± 17**.**85)**
(Time/s)	11.89	28.97	13.65	11.89	18.98	23.41
Lasso SRDA	**0**.**96 ± 0**.**01**	**0**.**95 ± 0**.**02**	**0**.**79 ± 0**.**03**	**0**.**96 ± 0**.**01**	**0**.**92 ± 0**.**02**	**0**.**81 ± 0**.**03**
(Angle)	**(135**.**17 ± 5**.**63)**	**(134**.**96 ± 11**.**08)**	(**135**.**10 ± 5**.**06**)	**(135**.**17 ± 5**.**63)**	**(134**.**64 ± 10**.**49)**	**(133**.**39 ± 4**.**87)**
(Time/s)	6.19	5.99	6.14	6.19	6.08	6.05
L1 SR (Angle)	**0**.**96 ± 0**.**01 (134**.**70 ± 5**.**11)**	0.94 ± 0.04 (130.72 ± 34.58)	**0**.**79 ± 0**.**03 (133**.**09 ± 4**.**98)**	**0**.**96 ± 0**.**01 (134**.**70 ± 5**.**11)**	**0**.**92 ± 0**.**03 (135**.**08 ± 10**.**12)**	0.80 ± 0.05 (134.39 ± 4.87)
(Time/s)	75.65	68.38	80.08	75.65	158.10	129.73
L1 GE (Angle)	**0**.**96 ± 0**.**01 (134**.**70 ± 5**.**11)**	**0**.**95 ± 0**.**02 (135**.**07 ± 7**.**44)**	**0**.**79 ± 0**.**02 (135**.**15 ± 20**.**87)**	**0**.**96 ± 0**.**01 (134**.**70 ± 5**.**11)**	**0**.**92 ± 0**.**02 (135**.**07 ± 9**.**04)**	**0**.**81 ± 0**.**03 (133**.**45 ± 17**.**85)**
(Time/s)	4.63	5.92	5.93	4.63	4.91	5.07

#### Effect of outlier strength

This simulation generates datasets contaminated by outliers with different strengths. The outlier is generated from a Gaussian distribution with fixed variance (0.5, 0.5) and varied mean from (6.00 6.00) to (8.60, 8.60), where the outlier strength ($6.00\times \sqrt{2}$ ~ $8.60\times \sqrt{2}$) can be adjusted by the varied mean, aiming to reveal the effect of the outlier strength on the hyperplane. We fix the number of outliers as 5% of the total sample number. The procedure is repeated 100 times, and the mean accuracies are reported in Table 1 on the right side. The mean effect of the outlier strength on the hyperplane is also given in Fig. 1 for all the classifiers. In this simulation study, we also do not report the corresponding hyperplane of SRKDA under various outlier strengths, as in the first simulation study.

### Actual Dataset

#### Gene Datasets

In real applications, gene data are usually contaminated by outliers due to measurement errors and random fluctuation at various manufacturing stages. Thus, in the following experiments, we tested the classification performance of the eight GE extensions on two gene datasets: One is a colon cancer dataset^35^ and the other is a Leukemia dataset^36^.

**Figure 2:**
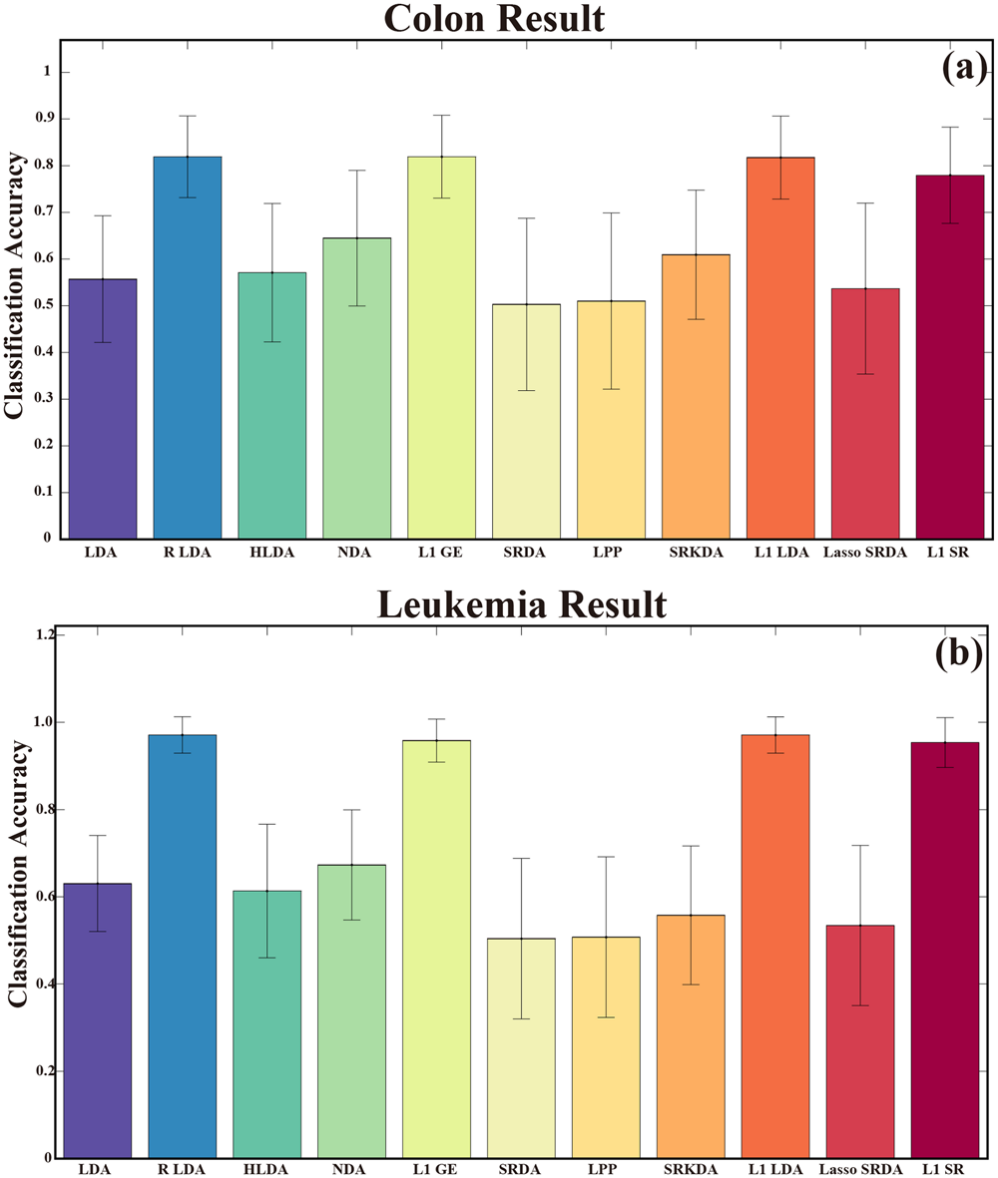
The gene dataset classification based on eight classifiers. (**a**) Colon cancer data. (**b**) Leukemia data.

The first colon cancer dataset contains the expression levels of 2000 genes taken in 62 different samples, i.e., a 62 × 2000 data matrix. For each sample, the matrix indicates whether it is from a tumor biopsy. Forty samples are positive, and 22 samples are negative. In our experiment, a 5-fold CV is adopted and repeated 100 times. In each 5-fold CV run, approximately 49 samples are included in the training set, and the 2000-length feature is reduced to a 49-length for each sample to serve as an input for classification according to previous studies^37,38^. The total time consumptions for the eight GE extensions to estimate the mapping information are 4.14 s, 1.08 s, 11.69 s, 35.30 s, 14.93 s, 0.90 s, 2.51 s, 0.91 s, 69.92 s, 2.17 s and 618.65 s. The mean accuracies of 100 runs for the eleven classifiers are given in Fig. 2(a).

The second leukemia dataset is a collection of expression measurements reported by Golub *et al*. (1999). The data set contains 72 samples. These samples are divided into two variants of leukemia: 25 samples of *acute myeloid leukemia* (AML) and 47 samples of *acute lymphoblastic leukemia* (ALL). The source of the gene expression measurements is taken from 63 bone marrow samples and 9 peripheral blood samples. The gene expression levels in these 72 samples are measured using high-density oligonucleotide microarrays. The expression levels of 7129 genes are reported. A 5-fold CV is used to evaluate the classification performance of the eight GE extensions on this dataset. In each 5-fold CV run, approximately 58 samples are included in the training set, and the 7129-length feature is reduced to a 58-length for each sample to serve as the input for classification. The times required for all the classifiers to estimate the mapping information are 5.25 s, 1.18 s, 14.84 s, 38.94 s, 17.91 s, 0.97 s, 3.12 s, 0.96 s, 62.05 s, 1.93 s and 637.33 s. The corresponding recognition accuracies for the classifiers are shown in Fig. 2(b). Both Fig. 2(a) and (b) consistently indicate that L1 GE, L1 SR, L1 LDA and RLDA have markedly better recognition performance than the other seven classifiers.

#### BCI Dataset

EEG is often contaminated by the noise from eye blinks or head movement, which largely influence the performance of EEG-based brain computer interfaces. In this section, we compare the performance difference of the eight GE extensions on two motor imagery datasets. The datasets used here are Dataset IVa of BCI competition 3 and a dataset recorded in our lab.

The first dataset consists of EEG signals recorded from five subjects using 118 electrodes. In each trial, a visual cue with respect to the motor imagery is shown for 3.5 s. Three types of images are presented, i.e., left hand, right hand and right foot. The tasks for the imagery of the right hand and the left hand are adopted to evaluate the algorithm performance in the current work. For the two tasks, the total number of EEG trials for each subject is 280, and the data are bandpass filtered between 0.05 and 200 Hz and down-sampled to 100 Hz. Following the reported results in^11,39,40^, the time interval between 0.5 s and 2.5 s after the trial onset is selected for task recognition, and the training trial and testing trial numbers used in previous studies^41^ are also adopted in the current work.

The second dataset is from the MI BCI system developed in our group, which consists of EEG data from 19 subjects. During the online experiment, the subjects were required to sit in a comfortable armchair in front of a computer screen, and they were asked to perform motor imagery with the left or right hand according to the instructions appearing on the screen. Motor imagery lasted for 5 s, followed by 5 s of rest. Fifteen Ag/AgCl electrodes covering the sensorimotor area were used to record the EEG, and the signals were sampled using 1000 Hz and bandpass filtered between 0.5 Hz and 45 Hz. Four runs on the same day were recorded for each subject, with each run consisting of 50 trials (i.e., 25 trials for each class), and there was a 3-minute break between two consecutive runs. The time interval between 0.5 s to 5 s after trial onset was selected for task recognition. The 50 trials in the first run were used for training classifiers, and the remaining 150 were used to test the performance of the classifiers. The experiment was approved by the Ethical Committee of the University of Electronic Science and Technology of China (UESTC). Informed consent was obtained from all participants, according to the Declaration of Helsinki.

For both datasets, a common spatial pattern (CSP)^42^ is adopted to extract the MI rhythm related features, and the 6 features corresponding to the 6 most discriminative CSP filters^43^ are used for task recognition. The corresponding classification accuracies for the 23 subjects are illustrated in Table 2, where L1 GE shows better performance than the other classifiers.

**Table 2:** Classification accuracy for the two BCI datasets.

BCI Dataset	Algorithms										
	LDA	R LDA	HLDA	NDA	SRDA	LPP	SRKDA	L1 LDA	Lasso SRDA	L1 SR	L1 GE
S1	**0**.**54**	0.52	0.52	**0**.**54**	0.53	**0**.**54**	0.50	**0**.**54**	0.52	**0**.**54**	**0**.**54**
S2	0.90	0.90	0.83	0.90	0.90	0.90	**0**.**93**	0.88	0.92	0.89	0.88
S3	0.93	**0**.**94**	0.81	0.93	**0**.**94**	0.93	0.92	0.93	0.92	**0**.**94**	0.91
S4	0.57	0.53	0.57	0.57	0.54	0.57	**0**.**62**	0.60	0.53	0.53	0.58
S5	0.70	0.70	0.67	0.70	0.70	0.70	0.72	0.69	0.72	0.70	**0**.**77**
S6	0.78	0.77	0.57	0.78	0.78	0.78	0.77	**0**.**79**	0.74	0.78	0.68
S7	**0**.**76**	**0**.**76**	0.49	0.75	0.75	**0**.**76**	0.73	0.75	**0**.**76**	0.75	**0**.**76**
S8	0.60	0.61	**0**.**80**	0.59	0.61	0.60	0.57	0.63	0.60	0.59	0.60
S9	0.80	0.82	0.60	0.84	0.82	0.82	**0**.**88**	0.80	0.84	0.84	0.80
S10	0.60	0.62	0.52	0.58	0.64	0.58	0.74	**0**.**80**	0.62	0.79	**0**.**80**
S11	0.61	0.62	**0**.**82**	0.61	0.61	0.61	0.59	0.57	0.60	0.60	0.66
S12	0.71	0.70	0.71	0.73	0.70	0.71	0.63	0.82	**0**.**87**	**0**.**87**	0.82
S13	0.58	0.61	0.58	0.58	0.61	0.58	0.61	0.70	0.75	0.73	**0**.**77**
S14	0.57	0.59	**0**.**75**	0.56	0.59	0.57	0.54	0.58	0.60	0.58	0.60
S15	0.58	0.58	0.52	0.58	0.58	0.58	0.53	0.80	0.58	0.55	**0**.**81**
S16	0.66	0.67	0.77	0.66	0.67	0.66	0.68	0.68	0.69	**0**.**79**	0.73
S7	0.98	0.98	0.55	0.98	0.98	0.98	**0**.**99**	0.98	0.98	0.98	0.92
S18	0.50	0.51	0.71	0.50	0.51	0.50	0.53	0.94	0.50	0.92	**0**.**98**
S19	0.52	0.52	0.47	0.52	0.52	0.51	0.50	0.52	0.52	0.52	**0**.**54**
aa	0.68	0.68	**0**.**70**	0.68	0.68	0.68	0.68	0.68	0.68	0.69	0.68
al	0.98	0.98	**1**.**00**	0.98	0.98	0.98	**1**.**00**	0.98	0.98	0.98	0.98
av	0.68	0.69	0.49	0.68	0.68	0.68	0.66	0.70	0.68	**0**.**72**	**0**.**72**
aw	0.75	0.81	0.74	0.75	0.79	0.75	0.74	0.78	0.88	**0**.**89**	0.84
ay	0.81	**0**.**83**	0.82	0.81	0.81	0.81	0.70	0.78	0.80	0.82	0.81
Mean	0.70 ± 0.14	0.71 ± 0.14	0.67 ± 0.14	0.70 ± 0.14	0.71 ± 0.14	0.70 ± 0.14	0.70 ± 0.15	0.75 ± 0.13	0.72 ± 0.15	0.75 ± 0.15	**0**.**76 ± 0**.**13**
Highest Acc#	2/24	3/24	5/24	1/24	1/24	2/24	5/24	3/24	2/24	6/24	**9/24**

#### UCI Dataset

To evaluate the generalization performance of L1 GE on other applications, we use nine binary-class datasets in UCI machine learning repositories to evaluate the algorithm performance; details of these nine datasets can be found in^44^. Specifically, in these nine datasets, five are from a clinical environment, i.e., heart disease data, breast cancer data, liver disorder data, SPECT data and thrombin data, while the remaining four datasets are from other applications.

For the purpose of our experiments, all of the features in each dataset are used for classification. We adopt the 5-fold CV proposed in^17^ for comparison, and the mean accuracies for 100 repetitions of CVs are reported in Table 3 for eleven different classification approaches. As shown in Table 3, L1 GE shows better performance on 5 datasets among the 9 tested datasets, giving the highest accuracy (84%) and approximately 1~5% accuracy improvement compared to the other approaches.

**Table 3:** The classification for the UCI dataset.

UCI Dataset	Algorithm										
	LDA	R LDA	HLDA	NDA	SRDA	LPP	SRKDA	L1 LDA	Lasso SRDA	L1 SR	L1 GE
Australian	**0**.**85 ± 0**.**03**	**0**.**85 ± 0**.**03**	0.68 ± 0.05	0.85 ± 0.03	**0**.**85 ± 0**.**03**	0.66** ± **0.03	0.56 ± 0.03	0.72 ± 0.06	0.68 ± 0.03	**0**.**85 ± 0**.**03**	**0**.**85 ± 0**.**03**
Time	0.30	0.08	6.80	1.12	0.12	1.80	2.96	50.75	0.27	436.02	4.68
BreastC	**0**.**95 ± 0**.**02**	0.94 ± 0.02	0.59 ± 0.05	**0**.**95 ± 0**.**02**	0.94 ± 0.07	0.89** ± **0.03	0.67 ± 0.02	0.87 ± 0.03	0.92 ± 0.02	**0**.**95 ± 0**.**02**	**0**.**95 ± 0**.**02**
Time	0.35	0.10	7.06	1.47	0.13	1.37	2.34	64.82	0.23	347.54	8.50
HeartD	0.83 ± 0.04	0.80 ± 0.05	0.57 ± 0.07	0.82 ± 0.05	0.80 ± 0.05	**0**.**84 ± 0**.**05**	0.82 ± 0.05	0.83 ± 0.05	0.83 ± 0.05	0.83 ± 0.05	**0**.**84 ± 0**.**05**
Time	0.13	0.07	2.13	0.61	0.09	0.36	0.37	18.20	0.22	126.06	1.76
IonoS	0.83 ± 0.05	0.83 ± 0.04	0.70 ± 0.06	0.83 ± 0.04	0.83 ± 0.04	0.83 ± 0.04	0.72 ± 0.04	0.83 ± 0.04	**0**.**90 ± 0**.**03**	0.83 ± 0.04	0.83 ± 0.04
Time	0.28	0.10	4.11	1.34	0.11	0.50	0.69	56.84	0.31	297.69	3.46
Liver	0.65 ± 0.05	0.65 ± 0.05	0.61 ± 0.07	0.66 ± 0.06	0.65 ± 0.06	0.57** ± **0.05	0.59 ± 0.06	0.66 ± 0.05	0.66 ± 0.06	0.66 ± 0.05	**0**.**67 ± 0**.**04**
Time	0.13	0.06	2.33	0.60	0.09	0.45	0.57	17.11	0.23	218.45	2.05
Sonar	0.89 ± 0.05	0.93 ± 0.04	0.53 ± 0.09	0.87 ± 0.05	0.94 ± 0.04	0.89** ± **0.05	**0**.**98 ± 0**.**05**	0.88 ± 0.05	0.73 ± 0.07	0.87 ± 0.05	0.93 ± 0.05
Time	0.53	0.15	4.20	2.38	0.10	0.52	0.49	20.50	0.30	371.93	6.57
SPECT	0.72 ± 0.06	**0**.**73 ± 0**.**05**	0.52 ± 0.07	0.69 ± 0.06	**0**.**73 ± 0**.**05**	0.73** ± **0.06	0.79** ± **0.06	0.71 ± 0.06	**0**.**73 ± 0**.**06**	0.72 ± 0.05	0.72 ± 0.06
Time	0.36	0.13	4.00	1.93	0.13	0.53	0.37	40.11	0.30	156.61	5.36
'Winequality'	0.89 ± 0.01	0.82 ± 0.01	0.79 ± 0.01	**0**.**93 ± 0**.**01**	0.82 ± 0.01	0.83** ± **0.01	**0**.**93 ± 0**.**01**	0.84 ± 0.01	0.77 ± 0.77	0.86 ± 0.03	**0**.**93 ± 0**.**01**
Time	1.85	0.16	194.81	6.15	0.22	585.56	264.16	321.20	1.31	1008.39	78.36
'Thrombin	0.84 ± 0.08	**0**.**88 ± 0**.**20**	0.80 ± 0.12	0.78 ± 0.12	0.51 ± 0.24	0.48** ± **0.04	0.68** ± **0.01	0.78 ± 0.15	0.50 ± 0.23	0.87 ± 0.05	0.86 ± 0.08
Time	0.54	0.35	1.02	7.00	0.23	0.35	1.17	5.71	0.71	41.94	2.16
Mean_Result	0.83 ± 0.03	0.83 ± 0.04	0.64 ± 0.07	0.82 ± 0.07	0.79 ± 0.09	0.78** ± **0.04	0.75** ± **0.04	0.79 ± 0.06	0.75** ± **0.06	0.83** ± **0.04	**0**.**84 ± 0**.**04**
Highest Acc#	2/9	3/9	0/9	2/9	2/9	1/9	2/9	0/9	2/9	2/9	**5/9**

## Discussion

The simulation study quantitatively evaluates the possible influence of outliers on the performances of GE extensions when they are applied to pattern recognition. In the first experiment, we studied the influence of the ratios between samples and outliers on the hyperplane estimation with the mentioned eleven classifiers. As shown in Table 1 on the left side, when the occurrence rate of an outlier is increased from 0% to 8%, the performances of ten linear classifiers are all lowered. But in terms of classification accuracies, L1 GE and the other L1 norm-based classifiers consistently show the better performance compared with the L2 norm-based GE extensions. In essence, the accuracy difference among the eleven classifiers is determinative by the hyperplane estimated for classification, and the hyperplane angle in Table 1 also confirmed that the hyperplanes estimated in the L1 norm space are less influenced by the introduced outliers and that their angles are closest to the theoretical 135 degrees. Specifically, Fig. 1 visually reveals the different effects of outlier occurrence rates on the eleven classifiers, where the hyperplane of all the classifiers are deflected under noise conditions. However, the hyperplanes estimated in the L1 norm space are more robust to outlier influence with less deflection than the other six linear classifiers. As for the non-outlier case (i.e., 0% occurrence rate), the eleven classifiers expected for SRKDA can find the boundaries to discriminate the two classes well. However, if a dataset is contaminated with outliers, the corresponding hyperplanes estimated in the L2 norm space are obviously biased toward the outliers, resulting in the misclassification of some samples. Compared to the classifiers estimated in the L2 norm space, although L1 GE and other L1 norm-based classifiers are influenced by outliers, their hyperplanes can still provide good discrimination ability (i.e., close to the diagonal 135 degree direction) for the two classes.

In the second experiment, we studied the influence of various outlier strengths on the classification hyperplane. Similar to the results in the first experiment, all the linear classifiers used in this experiment were influenced by outlier strength. As shown in Table 1 on the right side, when the value of the outlier varied from $6.00\times \sqrt{2}$ to $8.60\times \sqrt{2}$, their performances decreased. However, in terms of classification accuracy, L1 norm-based classifiers consistently show better performance than the traditional GE extensions. Similar to the results on the left side, the hyperplane angles in Table 1 on the right side confirm that L1 norm-based classifiers are robust toward the influence of outlier strength and that the angles of their estimated hyperplanes are closest to the theoretical value of 135 degrees. Specifically, the hyperplanes estimated from different outlier-strength conditions in Fig. 1 also reveal the robustness of L1 norm-based classifiers. Another point to note is that even a small outlier, e.g., $6.00\times \sqrt{2}$, will lead to a biased hyperplane estimated by the L2 norm-based GE extensions. Compared to the traditional GE extensions, although L1 norm-based classifiers are also influenced by the outliers, their hyperplanes can still provide good discrimination ability (i.e., close to the diagonal 135 degrees direction) for these two classes.

Considering the results in Fig. 1, we can see that the hyperplanes estimated by HLDA under outlier conditions show much more bias toward the ideal boundary compared to the other nine classifiers, which accounts for the relatively lower accuracies for this classifier in the simulation studies. Among the existing LDA variants, HLDA aims to extend LDA toward problems with more complex distribution other than the homoscedasticity distribution with equivalent variance^29,30,45^. The motivation of HLDA is to address heteroscedastic situations where the classes may have a distribution with differences in both the variance and mean, which indicates that this method needs to estimate these two parameters from the training samples^29^. However, when the training sample is contaminated by outliers, both the mean and variance are inaccurately estimated to fit a false sample distribution, which will finally induce the biased hyperplane. Similar to HLDA, NDA is also designed to solve the problems with unequal variance distribution. For NDA the critical step is to estimate the distance between samples in one class and their nearest neighbor centers from another class^30^. When the training samples are contaminated with outliers, the distance between the noised samples and their neighbor centers is usually larger than that of other original samples, which plays a dominant role in the between-class scatter estimation. Consequently, more outlier numbers will lead to a more biased hyperplane estimated by NDA. RLDA is developed to address the possible singular problem existing in the covariance matrix^31^, and SRDA combined with regularization operators can decrease the time and memory consumption by the Eigen decomposition of dense matrices. For this simulation study, the covariance matrix has relatively larger coefficients in the diagonal direction, thus indicating the non-singularity of the covariance matrix. Therefore, when small regularization parameters (*α* = 1.0 or *α* = 0.70) are added to the diagonal coefficients in RLDA and SRDA, they will have little effect on the covariance properties, resulting in the similar results observed for LDA, RLDA and SRDA in the simulation study. Nevertheless, the effect of different regularization strategies used in RLDA and SRDA on the classification performance can be revealed by the actual datasets when the covariance matrix may be close to singularity. As SRKDA estimated hyperplanes in the kernel space whose dimension is usually more than 2, they cannot be illustrated in Fig. 1 as other linear GE extensions. However, Table 1 showed that SRKDA performs similarly in resisting outlier influence. In essence, SRKDA and SRDA share the same parameter estimation strategy, which indicates that when the outlier influence cannot be restricted by the kernel space, SRKDA will also be influenced. It is worth noting that the Gaussian kernel seems to be less sensitive than the other LS-based GE extensions when both the strength and occurrence of the outlier increases, which may be attributed to the Gaussian space used for kernel construction. In fact, the kernel space reflects the interactions between different samples. For a Gaussian kernel, the weights of the outliers tend to be small values. When the outlier ratio is small, it will not influence the real neighbor relationship in the kernel space. However, when the outlier ratio increases, more kernel-components will tend toward small values, which will greatly influence the stability of the kernel matrix. In addition, the types and parameters of kernels will also have an effect on the performance of related methods. Thus, it might be cumbersome to find proper kernels and the corresponding parameters for different applications. LPP is proposed to yield a nearest neighbor structure in low-dimensional space similar to that in high-dimensional space by preserving the local structure^32^. When the value of the outlier is small, it will have less influence on the main neighbor structure, resulting in a relatively robust hyperplane as illustrated by the red line in Fig. 1(g). However, when the outliers are stronger, the neighbor structure measured by the Euclidean distance will be destroyed, and the biased mapping information as illustrated in Fig. 1(g) will be estimated. Evidently, these linear GE extensions are not inferred in the outlier problem. As a result, they are all sensitive to the outliers in our simulation studies. It is worth noting that both L1 norm-based classifiers and SRKDA consistently show good ability to compress the outlier effect. Compared with SRKDA, L1 GE, L1 LDA and L1 SR are nonparametric methods, which indicates their convenience in real applications.

Compared with the L2 norm-based GE extensions, all the L1 norm-based classifiers mentioned in this work performed better, as illustrated in Fig. 1(e),(h–j), which could be attributed to the robustness of the L1 norm space to outlier influence. In addition to the better performance, we also observe that the differences in the objective function will have an influence on the hyperplanes, although the four mentioned L1 norm classifiers estimated the parameters in the same norm space. Through Fig. 1, we can see that the hyperplanes estimated by L1 SR and L1 SRDA are almost the same as shown in Fig. 1(i) and (j), which may be attributed to the spectral regression model utilized in their objective function. Meanwhile, the hyperplanes estimated by L1 GE and L1 LDA also hold some similarity in different outlier conditions as shown in Fig. 1(e) and (h). In fact, with the coding matrix *H* defined for supervised learning in the current work, (11) can be transformed into an L1 norm-based DA structure that is similar to L1 LDA, and this similarity can account for their close performance as outlier restriction methods in the simulation study. Although L1 GE and L1 LDA have a similar DA structure under supervised conditions, there are some differences in the definition of the scatter matrix in the DA denominator, which accounts for the different classification results when applied to the actual datasets. In essence, our proposed L1 GE can be flexibly transformed into other L1 norm-based GE extensions (i.e., L1 LDA, L1 LPP, L1 PCA, etc.), with a variety of coding matrices *H*. We will explore this in further work.

In biomedical engineering, gene data are usually measured for cancer diagnosis. However, gene expression data sets usually have a large number of variables but with a small number of samples^46^ that require robust classifiers for the clinical diagnosis of diseases. RLDA is one of the popular methods for such a situation^20,28^, and the classification results for these two datasets demonstrate the efficiency of RLDA for gene recognition. For a gene dataset, specific problems are encountered such as information redundancy or a low signal-to-noise ratio (i.e., strong noise artifacts)^36,37,47^. The conducted comparison of the two gene datasets confirmed that L1 GE may also have a stable ability to address these problems and has the closest performance to RLDA. In gene datasets, we also observe that Lasso SRDA performs worse than the other L1 norm-based classifiers. This may be due to the objective function used in Lasso SRDA. In essence, the distance measurement of Lasso SRDA is still designed in L2 norm space although it imposed the L1 norm constraint on the parameter, which may indicate that when the dataset suffers from the curse of dimensionality and strong noise artifacts, the Lasso SRDA will be influenced, regardless of how strong the constraints that are imposed on the parameters.

In BCI applications, artifacts are the main cause of reduced signal quality. Artifacts can be caused by various factors, such as electrode wire movement in the signal recordings and measurement errors in biochemistry. These artifacts usually hold a strength that is several orders of magnitude larger than the signal of interest and appear as spike-like waveforms of very short periods. Such artifacts can usually be treated as outliers and must be eliminated before further study. In studies based on biomedical signals, the easiest way is to reject the contaminated samples. However, this is not preferable because it may lead to the loss of other useful information. Other favorable methods such as BSS can also be applied for artifact rejection. However, it is difficult to determine the extent to which the samples may be contaminated by outliers, and it is also not trivial to eliminate the artifact components by the BSS methods. In addition, there is a rare report about the application of graph embedding analysis in scalp EEG BCI. To investigate the feasibility of L1 GE in the EEG BCI application, we evaluate the performance on two independent BCI datasets (total 24 subjects). Table 2 shows that 9 of 24 subjects can obtain the highest accuracy when L1 GE is used, which is the highest ratio among the eleven classifiers. Moreover, L1 GE shows 1~9% accuracy improvement compared to the other ten classifiers. The results in Table 2 show the potential of L1 GE for actual BCI application.

The presence of outliers is encountered not only in biomedical signal recording or gene expression data but also in a variety of other clinical applications. To evaluate the generalization performance of L1 GE, we used 9 UCI datasets with 6 datasets from the clinical application. The results for the UCI dataset presented in Table 3 reveal that L1 GE has the highest recognition accuracy (84%). Table 3 also demonstrates that no classifier consistently shows the best performance across the 9 different UCI datasets and that L1 GE achieves the best performance for 5 of the 9 datasets, hence outperforming the ten other classifiers. In contrast to the conventional L2 norm-based GE and its extensions, L1 norm-based classifiers will utilize an iterative procedure, which results in the relatively higher complexity and also accounts for more time consumption.

The applications to various datasets demonstrate that the L1 norm-based GE extensions are robust in dealing with the artifact-influenced classification. Compared to conventional graph embedding based on the L2 norm structure, the developed L1 norm GE maximizes the dispersion in the L1 norm space, which can provide a more powerful ability to suppress artifact effects. In this work, we mainly evaluated the performance of L1 GE in supervised learning. For unsupervised learning, one crucial step is to estimate the symmetric factor *H*, which holds the structure information in similarity matrix *W*^48,49^. In essence, our proposed L1 GE can be flexibly transformed into other L1 norm-based GE extensions, with a variety of coding matrices *H*. In future work, we will explore the efficiency of L1 GE when it is transformed into other L1 norm-based GE extensions, and we will also do further analysis on its efficiency in unsupervised and semi-supervised learning.

## Methods

### Graph embedding

Define $m$ samples $X={\{{x}_{i},{x}_{i}\subset {R}^{n\times 1}\}}_{i=1}^{m}\subset {R}^{n\times m}$ from $C$ classes. In general graph embedding, each sample point can be treated as a vertex in an adjacency graph $G\in {R}^{m\times m}$, where *n* denotes the sample dimension. The corresponding edges in *G* represent a statistical relationship between each pair of these sample points. The motive of graph embedding is to represent each vertex of *G* in a lower dimensional space and preserve the original edge information between vertex pairs. Essentially, graph embedding estimates the response vector $y\in {R}^{m\times 1}$, which maximizes the following function:

1 $$J(y)=\frac{{y}^{T}Wy}{{y}^{T}Dy}\,\,$$

where *T* denotes the transpose and $W\in {R}^{m\times m}$ is a sparse symmetric matrix reflecting the weight of the joining edge between vertices *i* and *j* as

2 $${W}_{ij}=\{\begin{array}{c}1/{m}_{k},\,\,\,{\rm{if}}\,{x}_{i}\,{\rm{and}}\,{{\rm{x}}}_{j}\,{\rm{both}}\,\\ \,\,\,\,\,\,\,{\rm{belong}}\,{\rm{to}}\,{\rm{the}}\,k-\mathrm{th}\,\mathrm{class};\\ 0,\,\,\,\,\,\,\,\,{\rm{otherwise}}.\end{array}$$

and *m*_*k*_ is the sample number of the *k*-th class. *D* is a diagonal matrix whose entries are column or row sums of *W*^31^. Note that the scaling of the projection *y* will have no effect on the objective value. Thus, maximizing *J*(*y*) is tantamount to the following constrained optimization problem as

3 $$\{\begin{array}{c}\mathop{\text{arg}\,\max }\limits_{w}\,\,\,{y}^{T}Wy\\ subject\,to\,{y}^{T}Dy=1\end{array}\,\,\,\,$$

By introducing the Lagrange multiplier, the objective function can be rewritten as

4 $$L(y;\lambda )={y}^{T}Wy-\lambda ({y}^{T}Dy-1).$$

Taking the derivative of (4) with respect to *y* under the condition of $\partial L/\partial y=0$, response vector *y* can be estimated by using the generalized eigenvalue equation as

5 $$Wy=\lambda Dy\,$$

where $\lambda$ denotes the eigenvalue of the generalized eigenproblem, and *y* is the corresponding eigenvector. For multiple response vectors, the above equation (5) can be solved as

6 $${D}^{-1}WY={\rm{\Sigma }}Y$$

where *Y* is the matrix consisting of the eigenvectors of ${D}^{-1}W$, and ${\rm{\Sigma }}=diag({\lambda }_{1},{\lambda }_{2},\mathrm{...}{\lambda }_{m})$ is a diagonal matrix consisting of the eigenvalues of ${D}^{-1}W$. For classification purposes, there are only $(C-1)$ eigenvectors corresponding to the maximum $(C-1)$ eigenvalues. However, the response vectors ${\{{y}_{i}\}}_{i=1}^{C-1}\subset {R}^{m\times (C-1)}$ inferred from (6) only provide mapping information in the training set. To expand the mapping information for the testing sample, a simple way is to estimate some projections between the response vector and sample points. By replacing *y* with ${X}^{T}\alpha$, the objective function in (1) could be rewritten as

7 $$\mathop{\text{arg}\,\max \,}\limits_{\alpha }\,J(\alpha )=\mathop{\text{arg}\,\max }\limits_{\alpha }\frac{{\alpha }^{T}XW{X}^{T}\alpha }{{\alpha }^{T}XD{X}^{T}\alpha }$$

where $\alpha \subset {R}^{n\times 1}$ is the mapping projection between the defined graph and samples. Therefore, the optimal solution of equation (7) is a mapping $\alpha \subset {R}^{n\times 1}$, which can transform samples *X* to *Y* by preserving the manifold structure defined in *W* as much as possible. More details about graph embedding can be found in Appendix B.

### L1 Graph embedding

Noting that *W* is a symmetrical matrix and *D* is a diagonal matrix, equation (7) can be formatted as

8 $$\begin{array}{c}\mathop{\text{arg}\,\max }\limits_{\alpha }\,J(\alpha )=\mathop{\text{arg}\,\max }\limits_{\alpha }\frac{{\alpha }^{T}XW{X}^{T}\alpha }{{\alpha }^{T}XD{X}^{T}\alpha }\\ \,\,\,\,\,\,\,\,\,\,\,=\,\mathop{\text{arg}\,\max }\limits_{\alpha }\frac{{\alpha }^{T}XH{H}^{T}{X}^{T}\alpha }{{\alpha }^{T}X\sqrt{D}{\sqrt{D}}^{T}{X}^{T}\alpha }=\frac{{\Vert {\alpha }^{T}XH\Vert }_{2}^{2}}{{\Vert {\alpha }^{T}X\sqrt{D}\Vert }_{2}^{2}}\end{array}$$

where ${\Vert \bullet \Vert }_{2}$ denotes L2 norm, $W=H{H}^{T}$ and ${D}_{ii}={\sum }_{j=1}^{m}{W}_{ij}$. Because *W* is a symmetric matrix, there are several effective strategies to implement symmetric factorization so that we can easily estimate *H*^48,49^. As revealed in equation (8), the graph embedding is essentially derived from the L2 norm structure. However, the L2 norm has been proven to be prone to the presence of outliers^17^, which indicates that in practical applications, outliers will cause an unexpected effect on related analyses such as signal processing and feature extraction. To improve the robustness of parameter estimation in this framework, some schemes like sparse constraint with an L1 norm are proposed to alleviate the outlier effect^50,51^. However, most of these schemes are mainly focused on imposing restrictions on parameters but still leave the main structure of the objective function in the L2 norm space, which will inevitably exaggerate the outlier effect, regardless of how much these parameters are emphasized. Motivated by the merit of the L1 norm in suppressing the outlier effect, we proposed estimating the mapping denoted in (7) and (8) with the L1 norm space instead of the L2 norm as

9 $$\mathop{\text{arg}\,\max \,}\limits_{\alpha }\,\tilde{J}(\alpha )=\mathop{\text{arg}\,\max }\limits_{\alpha }\frac{{\Vert {\alpha }^{T}XH\Vert }_{1}}{{\Vert {\alpha }^{T}X\sqrt{D}\Vert }_{1}}$$

where ${\Vert \bullet \Vert }_{1}$ denotes the L1 norm. We refer to (9) as L1 Graph embedding (L1 GE). For pattern recognition, when all the training samples are labeled, we have ${\sum }_{k=1}^{C}{m}_{k}=m$. Suppose that all of the data points in *X* are ordered according to their labels as *X* = [*X*^(1)^, *X*^(2)^,…, *X*^(C)^,…, *X*^(k)^], where ${X}^{(k)}=[{x}_{1}^{(k)},{x}_{2}^{(k)},\mathrm{...},{x}_{{m}_{k}}^{(k)}]$ with ${x}_{i}^{(k)}$ being the *i*th feature vector for the *k*th class; then, the weight matrix *W* can be defined as a c-block diagonal matrix^31^, with each block being a symmetric matrix as

10 $$W=[\begin{array}{cccc}{W}^{(1)} & 0 & 0 & 0\\ 0 & {W}^{(2)} & 0 & 0\\ 0 & 0 & \ddots  & 0\\ 0 & 0 & 0 & {W}^{(C)}\end{array}]$$

where ${W}^{(k)}$ is an ${m}_{k}\times {m}_{k}$ matrix with all of the elements as $1/{m}_{k}$ and $D\in {R}^{m\times m}$ is a unit matrix. Let $\tilde{X}=X-\bar{X}$; we can rewrite (10) as

11 $$\mathop{\text{arg}\,\max }\limits_{\alpha }\,\tilde{J}(\alpha )=\mathop{\text{arg}\,\max }\limits_{\alpha }\frac{{\Vert {\alpha }^{T}{{\rm{\Phi }}}_{b}\Vert }_{1}}{{\Vert {\alpha }^{T}\tilde{X}\Vert }_{1}}$$

where $\bar{X}$ is the mean of $X$, and ${{\rm{\Phi }}}_{b}=\tilde{X}H$. $H\in {R}^{m\times c}$ is a coding matrix with columns indicating the class types and rows indicating the samples. The values in each column of *H* are defined as

12 $${H}_{k}={[\mathop{\underbrace{0,\mathrm{...},0}}\limits_{{\sum }_{i=1}^{k-1}{m}_{i}},\mathop{\underbrace{1/\sqrt{{m}_{k}},\mathrm{...},1/\sqrt{{m}_{k}}}}\limits_{{m}_{k}},\mathop{\underbrace{0,\mathrm{...},0}}\limits_{{\sum }_{i=k+1}^{c}{m}_{i}}]}^{T}.$$

In essence, by using the logarithm transformation, equation (12) is formatted as

13 $$J(w)=\mathop{\text{arg}}\limits_{w}\,\max \,\,\mathrm{ln}(||{\alpha }^{T}{{\rm{\Phi }}}_{b}|{|}_{1})-\,\mathrm{ln}(||{\alpha }^{T}\tilde{X}|{|}_{1}).$$

By introducing the sign function *R* and *Q*, we define the iterative direction for (14) as

14 $$d(\alpha (t))=\frac{(\sum _{i}^{C}{R}_{i}(t){{\rm{\Phi }}}_{b}(:,i))}{||\alpha {(t)}^{T}{{\rm{\Phi }}}_{b}|{|}_{1}}-\frac{(\sum _{j}^{N}{Q}_{j}(t)\tilde{X}(:,j))}{||\alpha {(t)}^{T}\tilde{X}|{|}_{1}},$$

where *R*(*t*), and *Q*(*t*) are defined as

15 $$\begin{array}{c}{R}_{i}(t)=\{\begin{array}{c}1,\,\alpha {(t)}^{T}{{\rm{\Phi }}}_{b}(:,i)\ge 0\\ -1,\alpha {(t)}^{T}{{\rm{\Phi }}}_{b}(:,i) < 0\end{array}\\ {Q}_{j}(t)=\{\begin{array}{c}1,\,\alpha {(t)}^{T}\tilde{X}(:,j)\ge 0\\ -1,\alpha {(t)}^{T}\tilde{X}(:,j) < 0,\end{array}\end{array}$$

In the above equations, ${{\rm{\Phi }}}_{b}(:,i)$ and $\tilde{X}(:,i)$ denote the *i*th column of matrices ${{\rm{\Phi }}}_{b}$ and $\tilde{X}$, respectively.

Based on the gradient in (14), the objective function in (11) can be solved by the iterations below:

1. Initialization. Set *t* = 0; Set the stop tolerance error ${\varepsilon }_{1}$ and ${\varepsilon }_{2}$ with a small positive number. Initialize $\alpha (0)\,$ with *N* random numbers and normalize it as $\alpha (0)\,=\alpha (0)/||\alpha (0)|{|}_{2}$.
2. Updating. Update the projection vector as $\alpha (t+1)\,=\alpha (t)+\eta d(\alpha (t))$ with $\eta$ being a small number accounting for the learning rate of iteration.
3. Convergence criterion. If $J(\alpha (t+1))-J(\alpha (t))|| < {\varepsilon }_{1}$, or ${\Vert \alpha (t+1)-\alpha (t)\Vert }_{2} < {\varepsilon }_{2}$, set $\alpha \,=\alpha (t+1)/||\alpha (t+1)|{|}_{2}$ and stop iteration; else, *t* = *t* + 1, and go to Step 2.

Using the above steps, the optimal $\alpha$ can be estimated. The computational complexity of L1 GE parameter estimation for a single iteration is $o(n\times (2m+2c+mc))$.

With the gradient in equation (14) and the iteration steps, the proposed objective function *J*(*α*(*t*)) is a non-decreasing function at each step of iteration *t*, which have been proved in Appendix A.

## Electronic supplementary material

Appendices
