# Supplementary material for: Improved Graph Embedding for Robust Recognition with outliers
Source: Sci Rep. 2018 Mar 9;8:4231. doi: 10.1038/s41598-018-22207-x (PMC5844917; doi:10.1038/s41598-018-22207-x)
Supplement: Supplementary file 1 — Appendices [file 41598_2018_22207_MOESM1_ESM.docx]

**Improved Graph Embedding for Robust Recognition with outliers**

Peiyang Li^a,b^, Weiwei Zhou^a,b^, Xiaoye Huang^a,b^, Xuyang Zhu^a,b^, Huan Liu^b^, Teng Ma^a,b^, Daqing Guo^a,b^, Dezhong Yao^a,b^, Peng Xu^a,b^,*

a The Clinical Hospital of Chengdu Brain Science Institute, MOE Key Lab for Neuroinformation, University of Electronic Science and Technology of China, Chengdu, China

b School of life Science and technology, center for information in medicine, University of Electronic Science and Technology of China, Chengdu, China

***Corresponding Author:**

Prof. Dr. Peng Xu

Address: #4, Section 2 of North Jianshe Road, Chengdu, Sichuan, China, 610054

Tel: +86-028-83206978, Fax: 86-028-83206978

E-mail: xupeng@uestc.edu.cn

Appendix A

In essence, the objective function proposed in our manuscript hold the upper bound, which can be written as

(1)

where$(\widetilde{X}^{-1})^{*}$denotes the pseudo-inverse matrix. With this upper bound and the iterative procedure proposed in our manuscript, the objection function$\widetilde{J}(w(t))$is a non-decreasing function at each step of iteration *t*. We can prove it as follows.

Proof. Considering equation (12) in the manuscript, at iteration *t*, we have that

$\widetilde{J}(\alpha(t))=\frac{\sum_{i=1}^{c} \left|\alpha^{T}(t)\Phi_{b}(:,i)\right|}{\sum_{j=1}^{m} \left|\alpha^{T}(t)\widetilde{X}(:,j)\right|}$ (2)

By using the definition of$R_{i}(t)$and$Q_{i}(t)$, equation (2) can be rewritten as

$\begin{matrix} \widetilde{J}(\alpha(t))=\frac{\sum_{i=1}^{c} \left|\alpha^{T}(t)\Phi_{b}(:,i)\right|}{\sum_{j=1}^{m} \left|\alpha^{T}(t)\widetilde{X}(:,j)\right|}=\frac{\sum_{i=1}^{c} \alpha^{T}(t)R_{i}(t)\Phi_{b}(:,i)}{\sum_{j=1}^{m} \alpha^{T}(t)Q_{j}(t)\widetilde{X}(:,j)} \\ =\frac{\alpha^{T}(t)\mu(t)}{\alpha^{T}(t)S_{\widetilde{X}}(t)\alpha(t)}=J^{*}(\alpha(t),\mu(t),S_{\widetilde{X}}(t)) \end{matrix}$ (3)

where$\mu(t)=\sum_{i=1}^{c} R_{i}(t)\Phi_{b}(:,i)$,$S_{\widetilde{X}}(t)=\frac{\sum_{j=1}^{m} (\widetilde{X}(:,j)\widetilde{X}^{T}(:,j))}{\left|\alpha^{T}(t)\widetilde{X}(:,j)\right|}$. For the convenience of the our proof, we introduce the following surrogate function

$L(\alpha,\mu,S_{\widetilde{X}})=\frac{\alpha^{T}\mu}{\alpha^{T}S_{\widetilde{X}}\alpha}$ (4)

as an intermediate step.

The gradient of $L(\bullet)$with respect to$\alpha(t)$can be represented as

$\begin{matrix} g(\alpha)=\frac{\partial L(\alpha,\mu,S_{\widetilde{X}})}{\partial\alpha} \\ =\frac{\mu\left[\alpha^{T}S_{\widetilde{X}}\alpha\right]-\left[\alpha^{T}\mu\right]S_{\widetilde{X}}\alpha}{\left(\alpha^{T}S_{\widetilde{X}}\alpha\right)^{2}}=\frac{\mu}{\left(\alpha^{T}S_{\widetilde{X}}\alpha\right)}-\frac{\left[\alpha^{T}\mu\right]S_{\widetilde{X}}\alpha}{\left(\alpha^{T}S_{\widetilde{X}}\alpha\right)^{2}} \\ =\frac{\mu\bullet\left(\alpha^{T}\mu\right)}{\left(\alpha^{T}S_{\widetilde{X}}\alpha\right)\bullet\left(\alpha^{T}\mu\right)}-\frac{\left[\alpha^{T}\mu\right]}{\left(\alpha^{T}S_{\widetilde{X}}\alpha\right)}\bullet\frac{S_{\widetilde{X}}\alpha}{\left(\alpha^{T}S_{\widetilde{X}}\alpha\right)} \\ =\frac{\left[\alpha^{T}\mu\right]}{\left(\alpha^{T}S_{\widetilde{X}}\alpha\right)}\bullet\left(\frac{\mu}{\left(\alpha^{T}\mu\right)}-\frac{S_{\widetilde{X}}\alpha}{\left(\alpha^{T}S_{\widetilde{X}}\alpha\right)}\right) \\ =\frac{\left\|\alpha^{T}\Phi_{b}\right\|_{1}}{\left\|\alpha^{T}\widetilde{X}\right\|_{1}}\bullet\left(\frac{\mu}{\left(\alpha^{T}\mu\right)}-\frac{S_{\widetilde{X}}\alpha}{\left(\alpha^{T}S_{\widetilde{X}}\alpha\right)}\right) \\ =\frac{\left\|\alpha^{T}\Phi_{b}\right\|_{1}}{\left\|\alpha^{T}\widetilde{X}\right\|_{1}}\bullet\left(\frac{\sum_{i=1}^{c} R_{i}\Phi_{b}(:,i)}{\sum_{i=1}^{c} \left|\alpha^{T}\Phi_{b}(:,i)\right|}-\frac{\sum_{j=1}^{m} \left|Q_{j}\widetilde{X}(:,j)\right|}{\sum_{j=1}^{m} \left|\alpha^{T}\widetilde{X}(:,j)\right|}\right) \end{matrix}$ (5)

Thus, the gradient value at the point$\alpha(t)$is obtained by substituting$\alpha(t)$into$g(\alpha)$:

$\begin{matrix} g(\alpha(t))=\frac{\left\|\alpha^{T}(t)\Phi_{b}\right\|_{1}}{\left\|\alpha^{T}(t)\widetilde{X}\right\|_{1}}\bullet\left(\frac{\sum_{i=1}^{c} R_{i}\Phi_{b}(:,i)}{\sum_{i=1}^{c} \left|\alpha^{T}(t)\Phi_{b}(:,i)\right|}-\frac{\sum_{j=1}^{m} \left|Q_{j}\widetilde{X}(:,j)\right|}{\sum_{j=1}^{m} \left|\alpha^{T}(t)\widetilde{X}(:,j)\right|}\right) \\ \propto\left(\frac{\sum_{i=1}^{c} R_{i}\Phi_{b}(:,i)}{\sum_{i=1}^{c} \left|\alpha^{T}(t)\Phi_{b}(:,i)\right|}-\frac{\sum_{j=1}^{m} \left|Q_{j}\widetilde{X}(:,j)\right|}{\sum_{j=1}^{m} \left|\alpha^{T}(t)\widetilde{X}(:,j)\right|}\right) \end{matrix}$ (6)

which is actually$d(\alpha(t))$in (14) in the manuscript. This indicates that$d(\alpha(t))$ is the vector that has the same direction (i.e., parallel to) as the ascending direction of$L(\bullet)$at$\alpha(t)$. Thus, we have

$\begin{matrix} \frac{\alpha^{T}(t)\mu(t)}{\alpha^{T}(t)S_{\widetilde{X}}(t)\alpha(t)}\leq\frac{\alpha^{T}(t+1)\mu(t)}{\alpha^{T}(t+1)S_{\widetilde{X}}(t)\alpha(t+1)} \\ \leq\frac{\alpha^{T}(t+1)\mu(t+1)}{\alpha^{T}(t+1)S_{\widetilde{X}}(t)\alpha(t+1)} \end{matrix}$ (7)

Note, the second inequality is due to the fact that$\left\{R_{i}(t+1)\right\}_{i=1}^{c}$is the set of optimal polarity corresponding to$\alpha(t+1)$such that, for all *i*,$\alpha^{T}(t+1)R_{i}(t+1)\Phi_{b}(:,i)\geq0$. However, $\left\{R_{i}(t+1)\right\}_{i=1}^{c}$cannot always guarantee the non-negative for$\alpha^{T}(t+1)R_{i}(t)\Phi_{b}(:,i)$.

To finish our proof, we need to further consider the denominator of (3). In this part, we would introduce the lemma: For any vector$v=(v_{1},...,v_{2})^{T}\in R^{n}$, the following equality holds ^53^,

$\left\|v\right\|_{1}=\min_{u\in R_{n}^{+}} \frac{1}{2}\sum_{k=1}^{n} \frac{v_{k}^{2}}{u_{k}}+\frac{1}{2}\left\|u\right\|_{1}$ (8)

For the convenience of proof, we set $\alpha^{T}(t)S_{\widetilde{X}}(t)\alpha(t)$as

$\frac{1}{2}\alpha^{T}(t)S_{\widetilde{X}}(t)\alpha(t)+\frac{1}{2}\left\|\alpha^{T}(t)\widetilde{X}\right\|_{1}$ (9)

we can infer that

$\begin{matrix} \alpha^{T}(t)S_{\widetilde{X}}(t)\alpha(t)=\frac{1}{2}\alpha^{T}(t)S_{\widetilde{X}}(t)\alpha(t)+\frac{1}{2}\left\|V\left(t\right)\right\|_{1} \\ =\frac{1}{2}\alpha^{T}(t)\left[\sum_{j=1}^{m} (\widetilde{X}(:,j)\widetilde{X}^{T}(:,j))/\left|V_{j}(t)\right|\right]\alpha(t)+\frac{1}{2}\left\|V\left(t\right)\right\|_{1} \\ =\frac{1}{2}\sum_{j=1}^{m} \frac{\left[\alpha^{T}(t)\widetilde{X}(:,j)\right]^{2}}{\left|V_{j}(t)\right|}+\frac{1}{2}\left\|V\left(t\right)\right\|_{1} \\ \Rightarrow\alpha^{T}(t+1)S_{\widetilde{X}}(t)\alpha(t+1)=\frac{1}{2}\sum_{j=1}^{m} \frac{\left[\alpha^{T}(t+1)\widetilde{X}(:,j)\right]^{2}}{\left|V_{j}(t)\right|}+\frac{1}{2}\left\|V\left(t\right)\right\|_{1} \\ \geq\min_{V\in R_{+}^{n}} \frac{1}{2}\sum_{j=1}^{m} \frac{\left[\alpha^{T}(t+1)\widetilde{X}(:,j)\right]^{2}}{\left|V_{j}\right|}+\frac{1}{2}\left\|V\right\|_{1} \\ =\frac{1}{2}\sum_{j=1}^{m} \frac{\left[\alpha^{T}(t+1)\widetilde{X}(:,j)\right]^{2}}{\left|V_{j}(t+1)\right|}+\frac{1}{2}\left\|V\left(t+1\right)\right\|_{1} \\ =\alpha^{T}(t+1)S_{\widetilde{X}}(t+1)\alpha(t+1) \end{matrix}$ (10)

Then, we have

$\frac{\alpha^{T}(t+1)\mu(t+1)}{\alpha^{T}(t+1)S_{\widetilde{X}}(t)\alpha(t+1)}\leq\frac{\alpha^{T}(t+1)\mu(t+1)}{\alpha^{T}(t+1)S_{\widetilde{X}}(t+1)\alpha(t+1)}$ (11)

Combining (7) and (11), we have

$\widetilde{J}(\alpha(t))\leq\widetilde{J}(\alpha(t+1))$ (12)

which establishes the proof.

Appendix B

Graph Embedding

Define $m$samples $X=\left\{x_{i},x_{i}\subset R^{n\times1}\right\}_{i=1}^{m}\subset R^{n\times m}$ from$C$classes. In general graph embedding, each sample point can be treated as a vertex in an adjacency graph$G\in R^{m\times n}$, where *n* denotes the sample dimension. The corresponding edges in *G* represent statistical relationship between each pair of these sample points. The motive of graph embedding is to represent each vertex of *G* in a lower dimensional space, and preserve the original edge information between vertex pairs. Essentially, graph embedding estimates the response vector$y\in R^{m\times1}$, which maximizes the following function

$J\left(y\right)=\frac{y^{T}Wy}{y^{T}Dy}$ (13)

where *T* denotes the transpose, $W\in R^{m\times m}$is a sparse symmetric matrix, reflecting the weight of joining edge between vertices *i* and *j* as

$W_{ij}=\left\{\begin{matrix} 1/m_{k}, \text{if} x_{i} \text{and} \text{x}_{j} \text{both} \\ \text{belong} \text{to} \text{the} k\text{-th} \text{class;} \\ \text{0,} \text{otherwise}. \end{matrix}\right.$ (14)

and *m_k_* is the sample number of the *k*-th class. *D* is a diagonal matrix whose entries are column or row sums of *W* ^31^. Note that the scaling of the projection *y* will have no effect on the objective value. Thus, maximizing *J*(*y*) is tantamount to the following constrained optimization problem as

$\left\{\begin{matrix} \mathop{\mathrm{argmax}}\limits_{w} y^{T}Wy \\ subject to y^{T}Dy=1 \end{matrix}\right.$ (15)

By introducing the Lagrange multiplier, the objective function can be rewritten as

$L\left(y;\lambda\right)=y^{T}Wy-\lambda\left(y^{T}Dy-1\right).$ (16)

Taking the derivative of (16) with respect to *y* under the condition of$\partial L/\partial y=0$, response vector *y* can be estimated by using the generalized eigenvalue equation as

$Wy=\lambda Dy$ (17)

where$\lambda$denotes the eigenvalue of the generalized eigenproblem, and *y* is the corresponding eigenvector. For multiple response vectors, the above equation (32) can be solved as

$D^{-1}WY=\Sigma Y$ (18)

where *Y* is the matrix consisting of the eigenvectors of$D^{-1}W$, and$\Sigma=diag\left(\lambda_{1},\lambda_{2},...\lambda_{m}\right)$is a diagonal matrix consisting of the eigenvalues of $D^{-1}W$. For classification purpose, there are only $(C-1)$eigenvectors corresponding to the maximum$(C-1)$eigenvalues. However, the response vectors$\left\{y_{i}\right\}_{i=1}^{C-1}\subset R^{m\times\left(C-1\right)}$inferred from (18) only provide mapping information in the training set. To expand the mapping information for the testing sample, a simple way is to estimate some projections between the response vector and sample points. By replacing *y* with$X^{T}\alpha$, the objective function in (13) could be rewritten as

$\mathop{\mathrm{argmax}}\limits_{\alpha} J\left(\alpha\right)=\mathop{\mathrm{argmax}}\limits_{\alpha} \frac{\alpha^{T}XWX^{T}\alpha}{\alpha^{T}XDX^{T}\alpha}$ (19)

where $\alpha\subset R^{n\times1}$ is the mapping projection between the defined graph and samples. Therefore, the optimal solution of equation (19) is a mapping$\alpha\subset R^{n\times1}$, which can transform samples *X* to *Y* by preserving the manifold structure defined in *W* as much as possible. Similar to the processes from (15) to equation (5) in the manuscript, the generalized eigenproblem for (19) is

$XWX^{T}\alpha=\lambda XDX^{T}\alpha.$ (20)

Equation (20) is usually called the spectral decomposition (SD). Obviously, the mapping projection $\alpha\subset R^{n\times1}$ is essentially the hyperplane for the classification ^31^.

To lower the time and memory burden when solving the generalized eigenproblem in (20), in ^14,15,26,31^, Cai et al solved the following regularized least squares problem to find$\left(C-1\right)$regression coefficient vectors $\left\{\alpha_{k}\right\}_{k=1}^{C-1}\subset R^{n}$ as

(21)

where$\mu$is a regularized parameter, which guarantees that the least squares problem is well-posed and has a unique solution. It is easy to infer that $\alpha_{k}$ is the solution of the linear equations system:

$\alpha_{k}=(XX^{T}+\mu I)^{-1}Xy_{k}.$ (22)

Although the regularized graph embedding defined in (22) can solve the problem of an ill-conditioned matrix, it is still constructed in the L2 norm space, which is also largely influenced by the outliers delivered into the variance matrix *XX^T^* ^54^.
